# Evaluation of the Tobii EyeX Eye tracking controller and Matlab toolkit for research

**DOI:** 10.3758/s13428-016-0762-9

**Published:** 2016-07-11

**Authors:** Agostino Gibaldi, Mauricio Vanegas, Peter J. Bex, Guido Maiello

**Affiliations:** 1 Department of Informatics, Bioengineering, Robotics and System Engineering, University of Genoa, Via All’Opera Pia, 13, Genoa, 16145 Italy; 2 Department of Psychology, Northeastern University, 360 Huntington Ave., Boston, MA 02115 USA; 3 UCL Institute of Ophthalmology, University College London, 11-43 Bath Street, London, EC1V 9EL UK

**Keywords:** Eye tracking, Low cost, Binocular, Eye movements, Saccade, Smooth pursuit, Vergence

## Abstract

The Tobii Eyex Controller is a new low-cost binocular eye tracker marketed for integration in gaming and consumer applications. The manufacturers claim that the system was conceived for natural eye gaze interaction, does not require continuous recalibration, and allows moderate head movements. The Controller is provided with a SDK to foster the development of new eye tracking applications. We review the characteristics of the device for its possible use in scientific research. We develop and evaluate an open source Matlab Toolkit that can be employed to interface with the EyeX device for gaze recording in behavioral experiments. The Toolkit provides calibration procedures tailored to both binocular and monocular experiments, as well as procedures to evaluate other eye tracking devices. The observed performance of the EyeX (i.e. accuracy < 0.6°, precision < 0.25°, latency < 50 ms and sampling frequency ≈55 Hz), is sufficient for some classes of research application. The device can be successfully employed to measure fixation parameters, saccadic, smooth pursuit and vergence eye movements. However, the relatively low sampling rate and moderate precision limit the suitability of the EyeX for monitoring micro-saccadic eye movements or for real-time gaze-contingent stimulus control. For these applications, research grade, high-cost eye tracking technology may still be necessary. Therefore, despite its limitations with respect to high-end devices, the EyeX has the potential to further the dissemination of eye tracking technology to a broad audience, and could be a valuable asset in consumer and gaming applications as well as a subset of basic and clinical research settings.

## Introduction

Eye-tracking technology provides a unique source of information about how humans and animals visually explore the world. Through eye tracking, we are able to investigate the cognitive processes underlying visual experience (e.g. attention, preference, discrimination), as well as to quantify the low-level parameters of oculomotor control (e.g. response latency, kinematics of eye movements). For these reasons, eye tracking technology is increasingly employed in a broad variety of research fields, from neuroscience to psychology, and has important clinical applications.

Originally, eye tracking research required invasive and uncomfortable techniques such as scleral search coils (Robinson 1963) or electro-oculography (Kaufman et al. 1993). Fortunately, the increase in computational power of standard PCs and graphic boards has fostered the development of less intrusive image-based techniques, including Purkinje image tracking, corneal reflection, iris and pupil tracking (see (Young and Sheena 1975; Jacob and Karn 2003; Canessa et al. 2012) for review). The rapid evolution of less intrusive, easy-to-operate eye tracking technology has led to systems that are now commonly employed in a variety of research and commercial projects (see (Duchowski 2007) for review). Indeed, eye tracking is now widely used in behavioral research across many different fields (see (Schötz et al. 2011) for review), from early vision and oculomotor research regarding saccades (e.g. (Collewijn et al. 1995; Jansen et al. 2009)), smooth pursuit (e.g. (Spering and Montagnini 2011)) and vergence eye movements (e.g. (Hung et al., 1994; Alvarez et al. 2002; Allison et al. 2004)) to higher cognitive tasks (attention, object recognition, spatial localization). Besides research tasks, eye movements are directly usable in practical human-computer applications (see (Duchowski 2002; Jacob and Karn 2003) for review) such as gaming (Corcoran et al. 2012) or human activity monitoring (Reimer and Sodhi 2006). Monitoring eye movements and rendering image content in a gaze-contingent fashion may also be beneficial for 3D virtual reality applications (Maiello et al. 2014).

Until very recently, eye tracking technology has been prohibitively expensive for anything other than industrial, clinical, or well-funded basic research, with the cost of an eye tracker ranging up to tens of thousands of dollars. However, as consumer demand drives down the cost of new technology and increases its availability in our daily lives, so has eye tracking technology begun to be inexpensive. Minimal eye tracking systems are now being embedded in smart-phones, and low cost eye tracking devices are beginning to appear on the market. Specifically two devices have attained the sub-$150 price point: the EyeTribe and the Tobii EyeX. The EyeTribe tracker has recently been evaluated for research purposes (Dalmaijer 2014; Ooms et al. 2015), while an evaluation of the Tobii EyeX is still needed.

The present work is focused on reviewing the capabilities of the Tobii EyeX Controller, which is a low price, image based eye tracking device from Tobii AB, conceived for consumer applications. The claims put forth by Tobii AB are that this new device is designed for eye gaze interaction with natural user experience where the user can sit, stand and move around somewhat freely. The Tobii EyeX can be mounted on both desktop and laptop setups, allowing for immediacy and ease of use. Moreover, the eye tracker is advertised as not requiring regular re-calibrations and as being able to cope with a great variety of physiological factors such as eye color, ethnicity, sight correction and age, independently of head movements and changing light conditions over time.

Tobii AB primarily produces research grade eye tracking devices. Although Tobii AB provides detailed technical specifications of all its research dedicated devices (see http://www.tobii.com/en/eye-tracking-research/global/products), an extensive description of the characteristics of the EyeX Controller is not available. In this paper, we provide an empirical analysis of the characteristics and technical specifications of the device, in terms of accuracy and precision, latency and sample frequency. In order to enhance the usability of the device for research (e.g. (Cornelissen et al. 2002)), we further develop and make available an open source Matlab Toolkit that can be used to interface with the eye tracker. The potential impact of the novel low-cost commercial technologies on research applications, as well as the widespread use of camera-based eye tracking methodology (e.g. see (Jacob and Karn 2003; Canessa et al. 2012; Svede et al. 2015; Xu et al. 2015)), promoted us to include, within the developed Toolkit, all procedures employed to test the Tobii EyeX, which can be easily adapted to other eye tracking devices. An interesting feature of the Tobii EyeX, that is not provided by the EyeTribe device, is the capability of providing a measure of the eye gaze separately for the left and the right eyes. In order to allow users to exploit the full potential of the Tobii EyeX Controller, we implemented a calibration procedure that can be carried out both binocularly and monocularly with each eye, and we present an evaluation of the differences between the monocular and binocular calibration procedures.

The present paper is organized as follows: ”The Tobii EyeX controller” describes of the characteristics of the controller, ”Materials and methods” provides an overview of the Matlab toolkit and of the proposed calibration procedures; “EyeX evaluation for research: results” reports the results obtained by the different calibration procedures, and provides examples regarding the capability of the controller in measuring saccade, smooth pursuit, vergence eye movements and fixation distributions in naturalistic viewing; finally in ”Discussion and conclusions” we discuss the strengths and limitations of the device based on our experimental evaluation.

## The Tobii EyeX controller

### Features of the system

The Tobii EyeX is an eye tracking device that allows moderately free head movements. It returns a real-time estimate of the left and right eye gaze positions on the screen, as well as the 3D position of the two eyes with respect to the screen center.

The actual technique exploited by the device for eye tracking is not declared by the manufacturer. Nevertheless, since the EyeX is based on Tobii’s latest hardware, it is reasonable to assume that it relies on the same techniques employed by the other Tobii eye tracking devices (e.g. X2-60 or TX300). These eye trackers are based on the pupil center and corneal reflection technique. The position of the pupil (which moves jointly with the eye) is computed with respect to the position of a glint (which is relatively invariant of the movement of the eye) produced by an infra-red illuminator on the cornea. The angular difference between the position of the pupil and the position of the glint is used to estimate the eye gaze. To ensure robust estimates of pupil location, both bright pupil and dark pupil eye tracking techniques are employed. In bright pupil eye tracking, an illuminator is placed close to the optical axis of the imaging device, causing the pupil to appear lit up. During dark pupil eye tracking, the illuminator is placed away from the optical axis, causing the pupil to appear black.

The image processing necessary for gaze data calculations is performed by the Tobii EyeX Engine, that runs on the PC to which the device is connected via USB3. Multiple applications can be connected as clients to the Tobii EyeX Engine over a LAN connection. These applications can be employed to perform a calibration of the gaze data and to gather the eye gaze data in real-time. The Tobii SDK released with the EyeX provides the libraries necessary to access the eye tracking data in the C/C++, C#/.NET, and Unity 3D programming languages. In order to enhance the accuracy of the gaze point estimation, the Tobii EyeX Engine provides a native calibration procedure (TNC) to be performed before the usage of the eye tracker by a new user. The procedure is required to compute the geometry of the setup (e.g screen size, distance, etc.) and to collect information about the light refraction and reflection properties of corneas of the subject.

### Technical specifications

Since the device is targeted for consumer applications, and not for scientific research, few technical specifications have been provided by the manufacturer. The **operating distance** refers to the minimum and maximum distances between a user’s eyes and the device at which eye tracking can be performed while maintaining robust tracking. The EyeX operating distance is specified at 450-800 mm. The EyeX allows for free head movements while a user’s head is located within a track box, which has the shape of a frustum with the vertex positioned in the center of the device. Thus, the allowable horizontal and vertical head movements change as a function of the distance of the user from the screen. For example, at a distance of 700mm users may move their head 240mm leftwards or rightwards and 195mm upwards or downwards. The maximum recommended screen size is 24 inches. Considering a user positioned at the far limit of the operating distance (800 mm), the working range of the device in degrees of visual angle is [−18°, 18°] on the *x*-axis, and [−10.5°, 10.5°] on the *y*-axis. The device will likely provide gaze estimates at wider gaze angles, to the detriment however of accuracy and precision, particularly in the corners of the monitor. The **sampling rate** of the device is the number of data samples per second collected for each eye, The Tobii EyeX has a nominal sampling rate of 60 Hz.

When employing an eye tracker for scientific research, a precise characterization of the spatial and temporal performance of the device is essential: the accuracy and precision of the gaze estimation need to be evaluated, as well as the system latency and the variability of the sampling rate. **Gaze accuracy** refers to the average angular error in gaze estimation when a user is fixating a known location in space. **Gaze precision** refers to the spread of the estimates of angular gaze position when the eyes are steady and fixating a target. Since the eye tracker can potentially be employed for gaze-contingent applications, in which stimuli on a computer monitor change as a direct result of changes in gaze position, the **system latency** can be defined as the delay between a change in gaze location and the related change on the display. This end to end latency consists of the exposure time of the eye tracker camera, the image read-out and transfer time, the image processing time, the data transfer time between the Tobii EyeX Engine and the end application, and the display refresh rate. The **sampling rate variability** can be evaluated by observing the distribution of the sampling rate estimates around the median observed sampling rate. A wide distribution indicates a high variability of the time interval between two consecutive eye position measurements.

In order to validate the Tobii EyeX for scientific research, we propose and perform a series of procedures to provide a quantitative evaluation of the spatial and temporal characteristics of the device.

## Materials and methods

### Matlab toolkit

To allow for a broader and more direct use of the Tobii EyeX device in scientific research, we have implemented a software Toolkit in Matlab which interfaces with the eye tracker controller. The Matlab Toolkit consists of four parts: 1) a client UDP (User Datagram Protocol) interface to connect Matlab with the Tobii server, 2) a set of basic connection functions for data transmission and reception, 3) a set of routines for standard use of the device, and 4) sample code provided to exemplify the usage of each function of the Toolkit in simple experiments in which we measure saccade, smooth pursuit, vergence and fixational eye movements. The graphical interface of the Toolkit has been implemented exploiting the Psychophysics Toolbox Version 3 (Kleiner et al. 2007; Brainard 1997; Pelli 1997). The client UDP interface has been developed via the Tobii Gaze SDK, thus allowing the Toolkit to be compatible with other eye tracking devices produced by Tobii such as the Tobii Rex and Tobii Steelseries Sentry.

The quality of eye tracking data in scientific experiments may be affected by both the subject and the operator (Nyström et al. 2013). Level of compliance to task instructions, variable environment illumination, glasses or contact lenses, makeup and eye physiology are all relevant factors with regards to the eye tracking data quality. To allow for a rapid online evaluation of gaze data quality, the Toolkit implements routines that resemble and extend the functionalities provided by the Tobii EyeX Engine. In particular, we provide routines to: 1) correctly position the user with respect to the screen; 2) calibrate both binocular and monocular gaze measurements, 3) visually check the outcome of the calibration. Moreover, we also release the code used to evaluate accuracy, precision and sample frequency of Tobii EyeX, which can be easily adapted to other eye tracking devices. A detailed description of the Matlab Tookit is provided in Appendix A.

#### Calibration procedure

When a normally-sighted observer is binocularly fixating a point in space, his/her optical axes are not always accurately aligned with the target. The angular misalignment, that can be both horizontal and vertical, is termed *fixation disparity*. While subjective measurements provide estimates of binocular fixation errors of up to 20 arc min, objective measurements have shown that binocular fixation errors can be considerably higher (Cornell et al. 2003). Considering a fixation disparity range between -30 and 120 arc min for near fixations, these misalignments are likely to affect the accuracy of the calibration procedure. It is well documented that the calibration procedure may greatly impact the quality of eye tracking data (Nyström et al. 2013). Furthermore, when dealing with binocular gaze data, the appropriate binocular calibration procedure must be carefully designed (Svede et al. 2015). The visual system has the tendency to weight the visual input from one *dominant* eye more than the other *non-dominant* eye (Nyström et al. 2013). Thus, when a subject is binocularly fixating, the dominant eye is pointing towards the intended target more accurately and precisely than the non-dominant eye, which ends up contributing more to fixation disparity (Simonsz and Bour 1991). To further complicate matters, eye dominance depends on gaze angle, so fixation disparity and monocular fixation accuracy will change as a function of gaze direction (Khan and Crawford 2001).

**Fig. 1:**
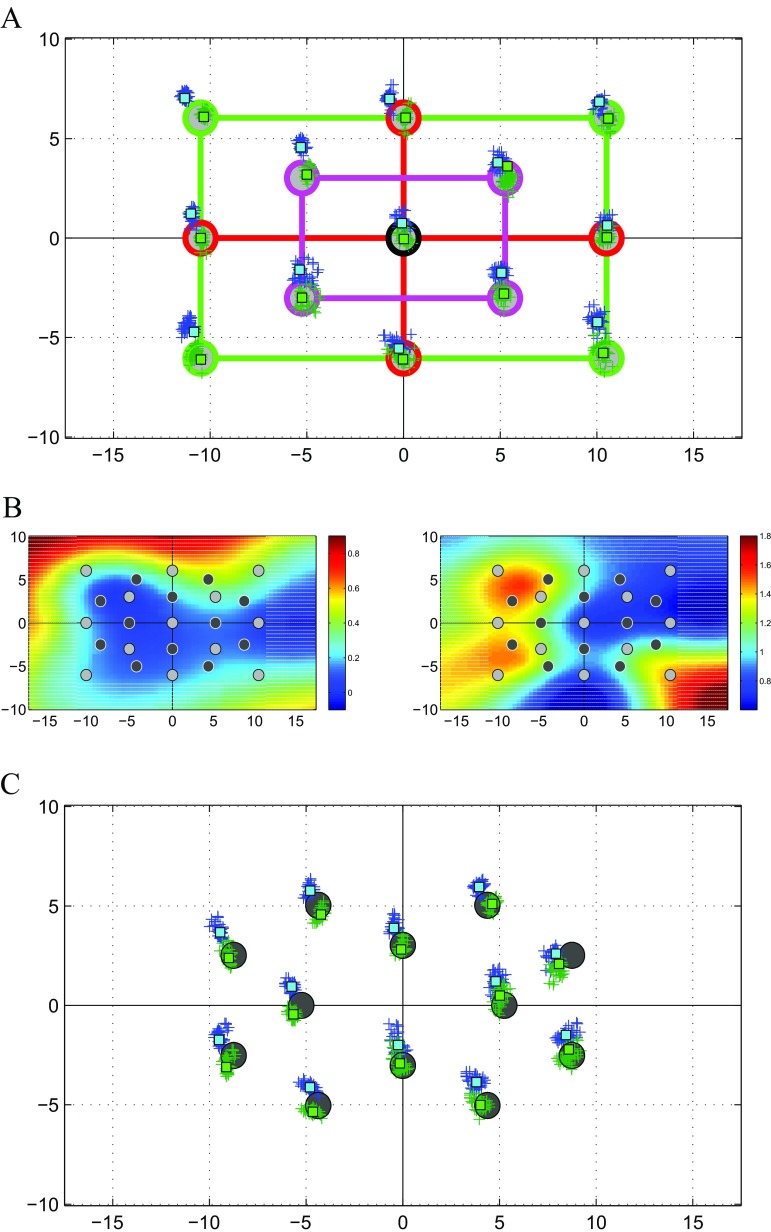
Example of the calibration procedure for the left eye in monocular viewing. **a** Calibration targets. The *circles* represent the angular target position [*d* *e* *g*] on screen for the proposed calibration procedure. The *red line* highlights the targets for the 5PC, the green line those added for the 9PC, and the *pink line* those added for the 13PC. *Blue* crosses represent the gaze direction measured by the Tobii EyeX calibrated via its native calibration routine. Green crosses represent the estimated gaze direction corrected with the 9PC. B. Calibration surface computed from the measured error on the horizontal (*left*) and vertical (*right*) axes, separately. The colormap represents the angular compensation to be applied to the gaze data acquired from the Tobii EyeX at each screen location. The *light and dark gray circles* are the targets used for calibration and testing, respectively, whereas the white *dashed* region indicates the screen area outside the calibration region. **c** Test targets. *Blue* crosses represent the gaze direction measured by the Tobii EyeX calibrated through it’s native calibration routine, *green* crosses represent estimated gaze direction corrected with the 9PC. The *circles* represent the angular target position [*d* *e* *g*] on screen, used for testing the calibration procedure

We investigated how to best tune the eye tracker calibration for binocular gaze data by implementing two separate calibration routines, one consisting of a single binocular procedure, and one consisting of two separate monocular procedures for the left and right eyes. The Tobii EyeX Engine provides a nine point calibration procedure in which the calibration points are positioned (see Fig. 1a) in the center of the screen (black circle), in the four corners (green circles), and at the four arms of a cross (red circles). The proposed thirteen point calibration procedure employs an additional 4 calibration targets (pink circles, Fig. 1a) in order to provide a finer coverage of the screen. These additional targets also allow us to evaluate the residual calibration error with greater spatial resolution.

During the calibration procedure, fixation targets are displayed in random order for two seconds each. The gaze data from the eye tracker are collected, and the initial 0.5 sec of data, which often correspond to compensatory saccades (Krauskopf et al. 1960; Cyr and Fender 1969), are discarded. Gaze position is computed as the median value of the data collected during the considered period, and the measurement error is computed as the distance between the displayed target and the measured gaze position on screen. Once all the targets have been displayed, an estimate of the error yielded by the device throughout the whole screen is computed separately for the *X* and *Y* axes. To avoid calibrating the device with data from potential misfixations, when the error at one or more target locations is greater than 0.5 deg, the calibration procedure is repeated at those target locations.

The measurement error is not uniform over the screen area (see Fig. 1a) but can be described as a three-dimensional surface. To estimate the error at every possible screen location, not solely at the calibration points, we exploited a surface fitting procedure. The error at each target position on the screen, computed during the calibration procedure, is fitted using a biharmonic function which provides a smooth surface interpolation (Carr et al. 2003; Ju et al. 2004). The fitting is performed separately on the X and Y coordinates (see Fig. 1b) of both left and right eye. Surface fitting is performed using a nonlinear least squares algorithm (Matlab^®^, The Mathworks, Natick, MA, USA). The resulting fitted surface can thus be employed to compensate for the residual error in real time on each single measurement provided by the eye tracker. Moreover, if gaze data are not employed interactively, the calibration can be applied to the recorded data offline, in post-acquisition processing.

Any successful calibration procedure, in addition to maximizing measurement accuracy, must also be non-fatiguing and user-friendly. Using fewer targets potentially allows for a shorter, more comfortable procedure. To evaluate the accuracy of calibration procedures with fewer number of targets, we considered subsets of the 13 point calibration data and tested (Fig. 1a): 1) a five point (5P) calibration (black plus green targets) , 2) a nine point (9P) calibration (black, red, and green targets), similar to the native one, and 3) a calibration considering the whole set of thirteen points (13P; black, red, green, and pink targets).

In order to evaluate the effectiveness of the calibration procedure, a different test set of 12 points was shown to the subjects, with the targets placed in different positions with respect to the calibration set. The spatial layout of these test targets can be seen in Fig. 1c. As for the calibration, the targets were shown in random order, both in binocular viewing and separately for the left and right eyes in monocular viewing. The data acquired when observers fixated these 12 test targets was corrected off-line using the calibration maps obtained from the 5P, 9P and 13P calibrations separately. This was done to evaluate the potential loss of accuracy when employing shorter calibration procedures.

### Data analysis

The residual error obtained with the different calibration procedures is reported as mean and standard deviation, separately for the dominant and the non dominant eye (see Table 1).

**Table 1:** Mean angular error [*d* *e* *g*]. Angular error (mean and standard deviation) across 15 subjects, computed separately for the dominant (D) and non-dominant (ND) eye, on the data calibrated with the TNC procedure and the proposed 5PC, 9PC and 13PC procedures. All possible couplings between monocular and binocular calibration and testing are presented: (MC-MT) monocular calibration and monocular test; (BC-BT) binocular calibration and binocular test; (MC-BT) monocular calibration and binocular test; (BC-MT) binocular calibration and monocular test. The table also reports significant *p*-values from all one-tailed, paired-sample *t*-tests comparing the TNC to all proposed calibration procedures

			TNC	5PC	9PC	13PC
D	MC-MT	*μ* ± *σ*	0.32 ± 0.12	0.30 ± 0.10	0.25 ± 0.09	0.24 ± 0.07
		p	-	< 5 × 10^−2^	< 10^−4^	< 10^−5^
	BC-BT	*μ* ± *σ*	0.31 ± 0.13	0.26 ± 0.09	0.23 ± 0.09	0.22 ± 0.08
		p	-	< 10^−3^	< 10^−6^	< 10^−6^
	MC-BT	*μ* ± *σ*	0.33 ± 0.15	0.29 ± 0.10	0.28 ± 0.12	0.27 ± 0.11
		p	-	< 10^−2^	< 10^−4^	< 10^−2^
	BC-MT	*μ* ± *σ*	0.31 ± 0.14	0.33 ± 0.13	0.28 ± 0.14	0.27 ± 0.13
		p	-	-	< 5 × 10^−2^	< 5 × 10^−2^
ND	MC-MT	*μ* ± *σ*	0.33 ± 0.15	0.32 ± 0.13	0.28 ± 0.10	0.23 ± 0.09
		p	-	-	< 10^−3^	< 10^−7^
	BC-BT	*μ* ± *σ*	0.34 ± 0.14	0.30 ± 0.10	0.30 ± 0.08	0.21 ± 0.09
		p	-	-	< 10^−7^	< 10^−9^
	MC-BT	*μ* ± *σ*	0.33 ± 0.11	0.34 ± 0.12	0.29 ± 0.10	0.27 ± 0.13
		p	-	-	< 10^−2^	< 10^−3^
	BC-MT	*μ* ± *σ*	0.34 ± 0.14	0.35 ± 0.18	0.29 ± 0.15	0.25 ± 0.13
		p	-	−	< 10^−3^	< 10^−5^

A paired one-tailed t-test was used to verify which of the proposed calibration procedures were significantly better than the Tobii naive calibration. The test was performed separately for the monocular and the binocular calibration procedures. P values < 0.05 were considered statistically significant and are reported in Table 1.

The Pearson’s correlation index was used to evaluate the repeatability of the proposed calibration, and the values are also reported as mean and standard deviation, separately for the dominant and the non dominant eye (see Table 2).

**Table 2:** Mean correlation index. Correlation index (mean and standard deviation) computed on each subject among the X and Y calibration functions obtained from the for repetitions of the calibration procedure. The index is computed on the X and Y functions, separately for the dominant (D) and non-dominant (ND) eye, considering 13PC, both monocularly and binocularly

	Dominant		Non-dominant	
	X (*μ* ± *σ*)	Y (*μ* ± *σ*)	X (*μ* ± *σ*)	Y (*μ* ± *σ*)
MONO	0.6923 ± 0.1311	0.5021 ± 0.1467	0.6805 ± 0.1098	0.5296 ± 0.1383
BIN	0.7355 ± 0.0872	0.5684 ± 0.1485	0.6900 ± 0.1378	0.5944 ± 0.1471

All statistical analyzes were performed with R software, version 3.0.1 (The R Foundation for Statistical Computing).

## EyeX evaluation for research: results

In the following we present an in depth evaluation of the Tobii EyeX device, of the developed Matlab Toolkit, and of the data that can be obtained from simple eye movement experiments.

### Device evaluation

In order to reliably measure the accuracy and precision of the device, we designed the following experimental setup. Observers were positioned at ≈700mm from the computer monitor with the head stabilized by a chin and forehead rest. The EyeX Controller was mounted at the bottom of the screen. Fifteen subjects participated in the experiment, all had normal or corrected to normal vision (i.e. they were wearing their prescription contact lenses). Eleven observers were right eye dominant, four were left eye dominant. The subjects underwent the monocular and binocular calibration and test procedures described in “Calibration procedure”. Each procedure was repeated four times per subject in random order. The experiments were run on a PC with an Intel Core i7-4700 CPU @2.40GHz, and 12GB of RAM, connected to a 17 inch LCD with 1920 × 1080 resolution at 60Hz, running on the Windows 7 Professional OS.

#### Accuracy and precision vs eccentricity

The performance of eye tracking devices may vary as a function of gaze angle away from straight ahead, central fixation. To evaluate the accuracy and precision of the EyeX device as a function of eccentricity away from central fixation, all raw data collected during the monocular and binocular test procedures were pooled together. The data were then separated with respect to the eccentricity of the visual target, computed as its angular distance from the center of the screen. This resulted in eight values of eccentricity, ranging from 0° to ≈12.2°. We observed that the angular error did not follow a Gaussian distribution, but was better described by a Poisson error distribution. Thus, rather than employing mean and standard deviation, we describe the performance metrics in terms of median and inter-quartile range. We therefore report accuracy as the distance between the median gaze estimate and the true target location. Precision is computed as the standard deviation of the estimates of angular gaze position when the eyes are steady and fixating a target.

**Fig. 2:**
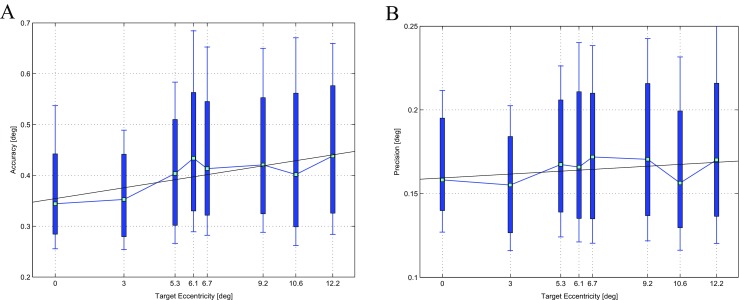
Distribution of Accuracy **(a)** and Precision **(b)** of the Tobii EyeX as a function of target eccentricity. Data are aggregated from across the fifteen observers and all calibration and test procedures. *Green squares* represent the median values, *thick blue* bars represent inter-quartile range, *blue* whiskers encompass minimum and maximum observed values, and the *black lines* are the linear regression lines passing through the median values

Figure 2 summarizes the results obtained regarding accuracy (A) and precision (B) of the Tobii EyeX as a function of visual angle. The device performs best at the center of the display. Accuracy worsens slightly at increasing eccentricities, whereas precision is approximately constant (*cf.* the linear regression lines). Accordingly, near the center of the monitor accuracy and precision can be considered to be < 0.4°, and < 0.2° respectively. At more than 5 degrees away from the center of the monitor, accuracy and precision worsen to < 0.6°, and < 0.25° respectively.

#### System latency and sampling frequency

To evaluate the device latency for gaze contingent applications, we employed a method similar to that described in Saunders and Woods (2014). We developed a simple gaze contingent display, consisting of two fixed targets and a cursor under the control of the user’s gaze. This simple gaze contingent display was implemented directly in C/C++ as well as with the Matlab Toolkit we developed (the Matlab Toolkit will be further evaluated in the following sections of this paper). We compared the C/C++ gaze contingent implementation against the Matlab gaze contingent implementation to assess whether the UDP server for data communication between Matlab and the Tobii EyeX Engine introduced any additional latency.

Observers were required to execute saccades back and forth between two targets presented on screen. Along with the saccade targets, the on-screen gaze position was displayed as a cursor in real time. While observers were performing the saccade task, we employed a high speed camera to record (at 240 fps) the PC screen and simultaneously the observer’s eye through a mirror. Two observers performed 20 saccades each while the camera simultaneously recorded both their eyes as well as the gaze contingent cursor on the screen.

After having acquired these video sequences, a video editing program (VSDC Free Video Editor) was used to perform a frame by frame inspection of the video sequences. The experimenter identified, for each saccade executed by the subjects, the movie frame in which the eye movement was initiated and the movie frame in which the gaze-controlled cursor began to move across the screen. The experimenter could then unambiguously count the number of frames between the actual eye movement onset and the corresponding response of the on-screen cursor. The total latency with which the system responded to the eye-movement was measured by multiplying the number of elapsed frames by the duration of each camera frame (4.2 ms). The estimated latency thus resulted from the sum of the display latency (hardware) and the gaze computation latency (software). The latency estimated from the data collected on both subjects with the C++ implementation was 48 ± 3 ms (mean ± standard deviation). The latency observed with the Matlab Toolkit was 47 ± 4 ms. These data confirm the reliability of the proposed procedure to estimate latency, since the uncertainty on the latency estimates is primarily due to the temporal resolution of the camera. Although different total latencies may be possible with different display or PC configurations (Saunders and Woods 2014), these data show that the UDP communication link between the Tobii server and Matlab does not appear to influence the system latency.

Because saccadic suppression (Volkmann 1962; Volkmann et al. 1968) or poor sensitivity to high speed retinal images (Dorr and Bex 2013) render a person visually insensitive for about 50 ms from the beginning and end of a saccade, the observed system latency is likely to go unnoticed by human users employing the system for gaze contingent applications.

The sampling rate and sampling variability were estimated from the data collected during the experiments performed to evaluate the calibration procedures, which provided a large quantity of samples. The observed sampling time of the system was 18.05 ± 2.49 ms (median ± inter quartile range), resulting in a median sample frequency of ≈55 Hz, which is slightly lower than the nominal frequency of 60 Hz.

The measurements we have just presented regarding latency and sampling frequency are necessarily system dependent. Thus, as a final consideration, we note that the use of a high performance PC and low-latency monitor are likely to improve the overall performance of the eye tracking system.

### Matlab toolkit evaluation

A detailed description of the implemented Matlab Toolkit is presented in the Appendix A. Here we focus on evaluating the calibration procedures we propose and implement in the Toolkit with regards to the accuracy of the gaze measurements.

#### Comparison between proposed calibration procedure and native Tobii calibration procedure

**Fig. 3:**
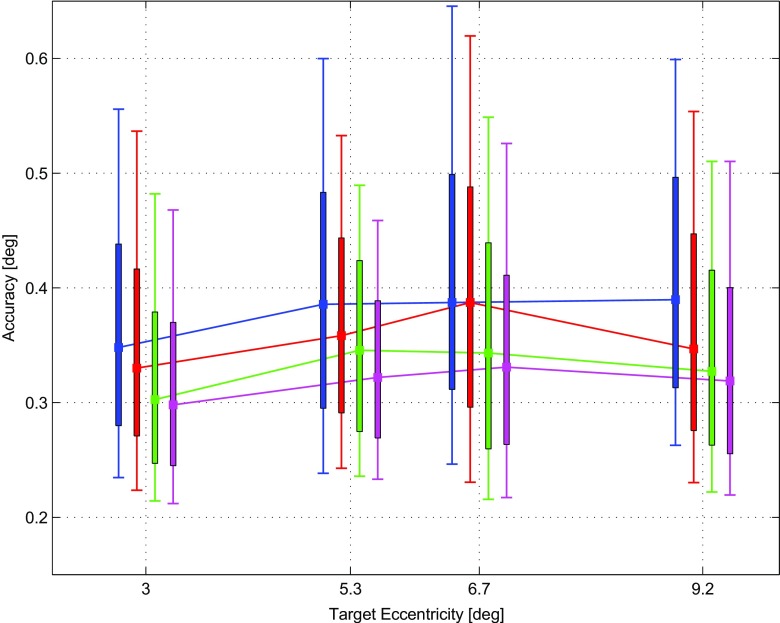
Angular error (accuracy) as a function of target eccentricity. The angular error has been measured at the test targets for the data calibrated via the TNC *(blue)*, 5PC *(red)*, 9PC *(green)* and 13PC *(pink)*. Squares are medians, thick vertical bars are the inter-quartile range, whiskers are the minimum and maximum values of the error distributions

In order to evaluate the influence of the proposed calibration procedures on the accuracy of the gaze measurements, we further analyzed the data collected as described in “Device evaluation”. We computed the angular error from the data obtained in the test procedure following the TNC, and on the same data corrected with the proposed 5PC, 9PC and 13PC. In Fig. 3 the accuracy for each calibration procedure is plotted as a function of angular distance from the screen center.

The 5PC (red) performs as well as or better than the TNC (blue). The 9PC and 13PC routines consistently outperform the TNC at every target eccentricity.

**Fig. 4:**
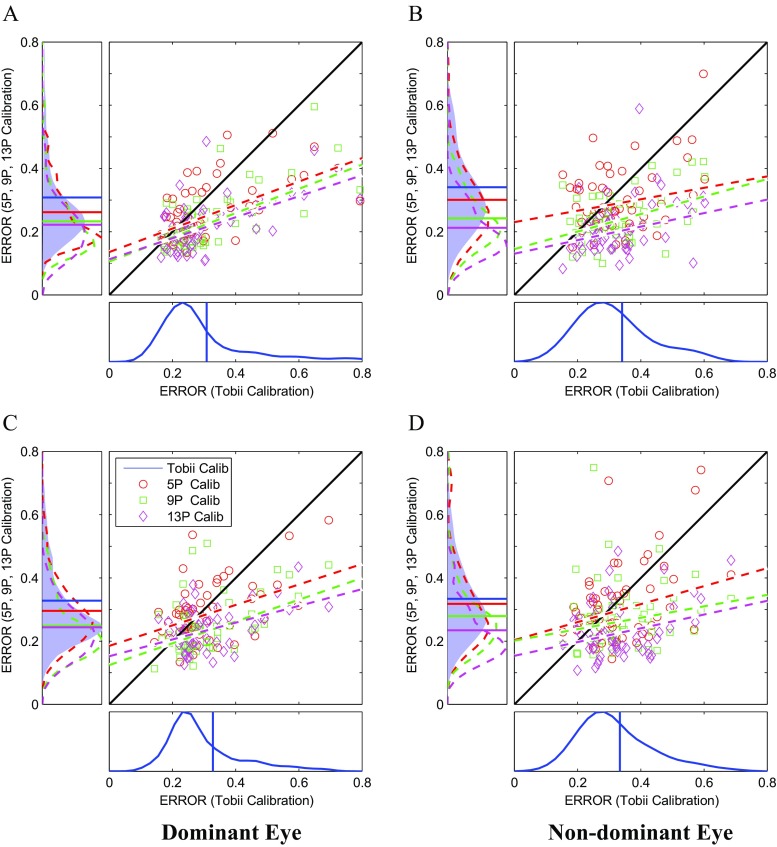
Mean calibration error. Scatter plots of the angular error comparing the TNC to the proposed calibration procedures, distinguishing between the dominant (**a and c**) and non-dominant (**b and d**) eye, and between a binocular test following a binocular calibration **(a–b)** and a monocular test following a monocular calibration **(c–d)**. Each sub-figure presents the scatter plots and the linear regression lines of the error of the TNC (*x*-axis) plotted against (*y*-axis) the error of the 5PC (*red circles*), 9PC (*green squares*) and 13PC (*magenta diamonds*). The insets below each figure show the distribution (*blue curve*) and median (*vertical line*) of the error observed with the TNC. The insets to the left of each figure show the distributions (*dotted curves*) and medians (*horizontal lines*) of the error observed with the proposed calibration procedures overlaid onto the TNC error distribution, represented by the *shaded blue* region for direct comparison

Moreover, these data were analyzed separately for the dominant and the non-dominant eye. Figure 4 shows the scatter plots of the angular error achieved by the TNC (*x-*coordinates) compared to the residual error (*y-*coordinates) after the 5PC (red), 9PC (green) and 13PC (pink). In order to evaluate the trend on the error, the data were fitted with a linear regression line. The horizontal inset represents the histogram of the error computed on the original data calibrated with the TNC, while the vertical inset is the same error computed on the data corrected by the 5PC, 9PC and 13PC. The histograms were computed via kernel density estimation (Botev et al. 2010). The median values are represented on the horizontal inset with a vertical bar, and on the vertical inset with horizontal ones. The figure provides an in-depth characterization of the effect of the different calibration procedures on the accuracy of the gaze measurements.

The histograms show that the error produced by the TNC (blue), as anticipated in “Device evaluation”, has a distribution which is skewed to the left, with a long right tail. These error distributions are well approximated by a Poisson distribution. As expected, the non-dominant eye (see Fig. 4, right column) is characterized by a larger mean gaze error and a wider error distribution with respect to the dominant eye (left column).

All three proposed calibration procedures reduce the mean error, especially at the right tail of the error distribution. This suggests that the proposed calibration procedures have the strongest effect on large errors. The linear regression highlights how the 5PC calibration, which relies on calibration points positioned away from the center of the monitor, reduces large errors at the borders of the monitor, but exacerbates small errors near the center of the display. Conversely, the 9PC (green) and 13PC (pink) procedures, which rely on a finer tiling of the workspace, are able to reduce both small and large errors. Accordingly, the histograms of the error distributions produced by the 9PC and 13PC are characterized by a narrower peak with respect to both the TNC and the 5PC, and by smaller median error values. These results are further confirmed by the regression lines passing through the data calibrated via the 9PC and 13PC. These regression lines fall below the diagonal throughout the error range, demonstrating that the errors are globally reduced.

These data have been further summarized in a table reporting the values of the angular error (mean and standard deviation) computed over the whole dataset (fifteen subjects, four repetitions, twelve test points, see Table 1). The statistical significance of the possible improvements has been assessed using a one-tailed paired-sample *t*-test, performed between the error produced by the TCP and the error produced by the proposed procedures. Consistent with what we have reported so far, the 5PC only occasionally significantly improved measurement accuracy. Conversely, the 9PC and 13PC always resulted in a statistically significant improvement of the gaze measurement accuracy. As expected, the 13PC, which relies on a larger number of calibration points, outperforms all the other procedures. Accordingly, the proposed procedure has been demonstrated to be equivalently effective in both the monocular and the binocular approaches.

As a final remark it is worth noting that the gaze measurement for the non-dominant eye suffers from larger measurement error with respect to the dominant one (*p* < 10^−3^). In agreement with a very recent study (Svede et al. 2015), this results strengthens the notion that careful choice of the appropriate calibration procedure is a mandatory step to increase the accuracy of binocular eye tracking data.

#### Comparison between single binocular calibration and two independent monocular calibrations for each eye

A further analysis was performed in order to highlight potential differences between monocular and binocular calibration procedures. Depending on the goal of an experiment or application, a binocular calibration might be better suited than a monocular one. For instance, when tracking the point of regard on a 2D screen, as in human computer interaction and gaming (Smith and Graham 2006; Dorr et al. 2007; Sundstedt 2012) or visual attention studies (Hoffman and Subramaniam 1995; Rayner 2009), a binocular calibration might be more appropriate than a monocular calibration. Conversely, if an experimental setup requires precise measurements of the position of each eye, which would be necessary when measuring vergence eye movements or the point of regard in three dimensional space, two separate monocular calibrations, one for each eye, are potentially preferable (Cornell et al. 2003; Gibaldi et al. 2015; Svede et al. 2015; Gibaldi et al. 2016).

In view of the above considerations, we evaluated the effect of performing two independent monocular calibrations and then performing a binocular test, as well as the effect of performing a single binocular calibration and then testing monocularly. The results have been summarized for the three calibration procedures in Table 1. The results show that mixing the couplings between monocular and binocular calibration and testing affects the accuracy of the gaze measurements. A careful inspection of Table 1 shows that data accuracy is best when the test is performed the same way as the calibration (i.e. a monocular test is used with a monocular calibration or a binocular test is used with a binocular calibration). In fact, in most of the measurements in which the monocular/binocular coupling between calibration and test was not preserved, accuracy was worse with respect to the corresponding “correct” coupling (*p* < 10^−2^ for 9PC and 13PC). Moreover, the mixed coupling results in a significant increase (*p* < 10^−4^) of the error variability in all the measurements.

The case of two monocular calibrations and subsequent binocular testing is particularly interesting: the loss in accuracy in this case is attributable to effects of eye dominance (as discussed above), so even though the accuracy might seem lower, the measurements might be closer to what the experimenter is truly interested in studying (e.g. fixation disparity (Svede et al. 2015)). Defining the appropriate calibration procedure is thus of paramount importance when designing an eye movement study. Within our Toolkit we thus provide the necessary tools to implement the appropriate procedure.

#### Repeatability of the calibration procedure

The repeatability of the calibration was evaluated from the data collected on the fifteen subjects by computing Pearson’s correlation index between the calibration functions obtained repeating the 13PC procedure four times. Each function was sampled over the screen area covered by the calibration procedure (see Fig. 1b), and the correlation index was computed between each possible coupling of the functions obtained from the four repetitions (i.e. 6 correlation estimates per subject). Table 2 reports mean and standard deviation of the correlation computed across the six estimates and fifteen subjects separately for the monocular/binocular calibration procedures and for the dominant/non-dominant eye. Whereas the calibration functions from different subjects were uncorrelated, the calibration functions from the same subject were consistently correlated independently of tested eye or monocular/binocular procedure (all *ρ* > 0.5), confirming the repeatability of the calibration procedures.

### Eye movement data quality

We have so far shown that the EyeX controller can be successfully employed via the MATLAB framework, and that the device, accessed through the Toolkit we provide, can be calibrated and employed for simple gaze-contingent applications, given the reasonably short system latency. Next, we verify whether it is possible to successfully measure the most common types of eye movements that are typically studied in basic and clinical research settings.

#### Saccade dynamics

To bring our high resolution fovea onto targets of interest preselected with our low resolution peripheral vision, our oculomotor system continuously makes fast, ballistic eye movements called saccades. Saccades are perhaps the most investigated type of eye movement, thus we devised a simple experiment to verify whether we could successfully measure simple saccadic eye movements.

*Experimental setup* The experiment was run on a standard PC equipped with Windows 7 Professional, with an Intel Core i7-4700MQ CPU @2.40GHz, and 12GB of RAM, with a 28 inch LCD with 1920 × 1080 resolution running at 60 Hz. Observers were positioned ≈500mm from the monitor, which subtended 70 × 40 degrees of visual angle. Observers were positioned in a chin and forehead rest to stabilize head movements. A 13 point calibration procedure was performed for each observer. The EyeX eye tracker was positioned below the monitor in front of the observers.

*Stimulus presentation* Observers were instructed to fixate a central red fixation dot presented on a uniformly black screen, and when ready, were required to initiate a trial by pressing a key on the keyboard in front of them. The fixation target would then turn white, and, after a 500 ms delay, the target would jump 10 degrees left. Observers were simply required to visually track the target as accurately as possible. The target would remain at the eccentric position for 750 ms, and then turn red once again and return to the center of the monitor. Each subject performed 50 eye movement trials.

**Fig. 5:**
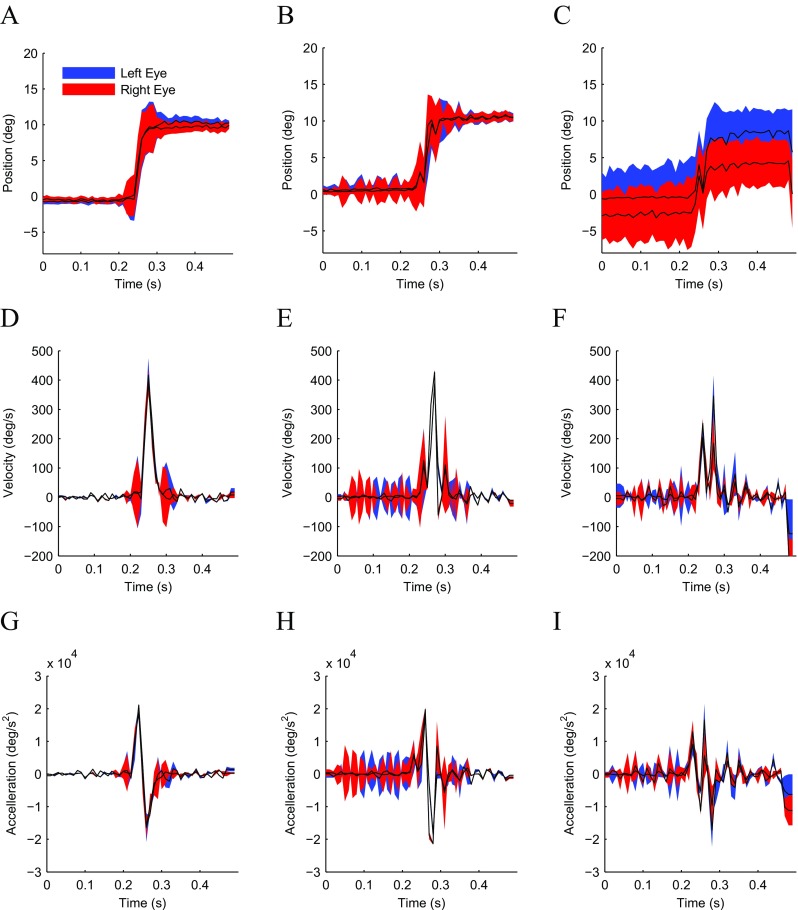
Stereotypical saccade dynamics. Saccade dynamics measured for *left (blue)* and *right (red)* eye in three subjects (columns) for 10 degree horizontal saccades. **a–c** Horizontal eye position as a function of time from saccade target onset. **d–f** Horizontal eye velocity. **h–i** Horizontal eye acceleration. The first subject **a,d,g** was author GM and an experienced observer. The second **b,e,h** and third **c,f,i** subjects were inexperienced naive observers. Data are the average from 50 trials. Shaded region represent ±1 SD

*Results* Figure 5 shows the results of our measurements of saccade dynamics in three observers. The first subject was an experienced observer (author GM), while second and third subject were naive observers. Figure 5a-c present average horizontal eye position as a function of time from target step for the saccades measured in all three subjects. As can be seen from the shaded regions representing the variability in the measurements, the data collected on the first two subjects (Fig. 5a, b) were highly reliable and accurate, whereas the data collected on the third subject (Fig. 5c) were more variable and particularly less accurate for the subject’s right eye (red trace) than for the subject’s left eye (blue trace). The saccades in all three subjects were initiated between 200-250 ms after the onset of the eccentric target, which is consistent with typical saccade latencies observed in the literature (Saslow 1967; Cohen and Ross 1977). The duration of the saccades was ≈50ms, which is also highly consistent with the literature on similarly sized saccades (Baloh et al. 1975; Bahill et al. 1981; Behrens et al. 2010).

Saccade velocity and saccade acceleration profiles are eye movement characteristics often investigated in the literature. We measured velocity (Fig. 5d-f) and acceleration (Fig. 5g-i) by taking the first and second derivative of the data in Fig. 5a-c using a two point differentiator. Qualitatively, reasonable velocity and acceleration profiles are observable in all subjects. Peak velocity was ≈400*d* *e* *g*/*s*, whereas peak acceleration and deceleration were ≈18000*d* *e* *g*/*s* ^2^, all values highly consistent with previous measurements of these parameters in normally sighted subjects (Bahill et al. 1981).

#### Smooth pursuit eye movements

Another commonly investigated class of eye movements are smooth pursuit eye movements, which allow us to closely track moving objects. We thus set out to verify whether we could reliably measure smooth pursuit eye movements with the Tobii ExeX in another simple experiment.

*Experimental setup* As in the previous experiment, we employed a standard PC, equipped with Windows 7 Professional, with an Intel Core i7-4700MQ CPU @2.40GHz, and 12GB of RAM, with a 28 inch LCD with 1920 × 1080 resolution running at 60 Hz. Observers were positioned ≈500mm from the monitor, which subtended 70x40 degrees of visual angle. Observers were positioned in a chin and forehead rest to stabilize head movements, and a 13 point calibration procedure was performed for each observer.

*Stimulus presentation* Observers were instructed to fixate a central red fixation dot presented on a uniformly black screen, and when ready, were required to initiate a trial by pressing a key on the keyboard in front of them. The fixation target would then turn white, and, after a 500 ms delay, the target would begin to move at a constant speed of 10 *d* *e* *g*/*s* to the right . After one second, the direction of the target would reverse and the target would return to the center of the monitor. Observers were simply required to visually track the target as accurately as possible. Once the target had returned to the starting position, it would turn red and a new trial could be commenced. Each subject performed 50 eye movement trials.

**Fig. 6:**
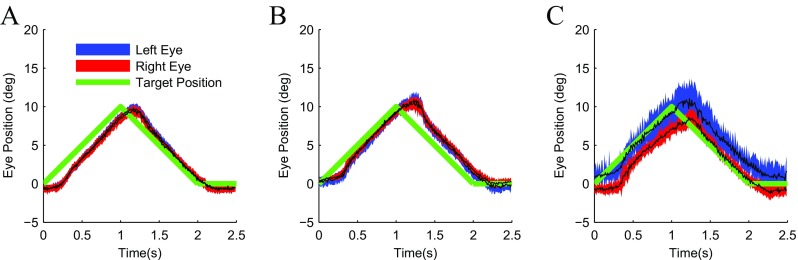
Stereotypical pursuit traces. Pursuit eye movements measured for *left (blue)* and *right (red)* eye in three subjects **(a–c)** for smooth eye movements in pursuit of a target (green trace) moving at 10 deg/s from the center of the screen to ten degrees right of center and back. First subject **a** was author GM and an experienced observer. Second **b** and third **c** subject were inexperienced naive observers. Data are the average from 50 trials. Shaded region represent ±1 SD

*Results* Figure 6 shows the results of our measurements of smooth pursuit eye movements in the same three observers as the previous experiment. As in the saccade experiment, the data collected on the first two subjects (Fig. 6a, b) were highly reliable and accurate, whereas the data collected on the third subject (Fig. 6c) were more variable. The typical characteristics (Robinson 1965; Spering and Montagnini 2011) of smooth pursuit eye movements can nonetheless be clearly observed in the data from all three subjects. In the initial *open-loop* stage of the tracking eye movement, after a latency ranging from 100-300 ms, the eyes accelerate and perform catch up saccades to capture the target. Then, in the *closed-loop* phase of the tracking eye movement, the eyes of the observers match the position of the moving target quite closely by maintaining the same speed as the target. When the target abruptly changes direction of motion, once again the eyes of the observers catch up and then match the smoothly moving target.

#### Vergence eye movements

When looking at an object binocularly, our two eyes must rotate in opposite directions to be correctly pointed towards the object. These disconjugate rotatory movements are called vergence eye movements. Vergence eye movements correctly position the retinal areas with highest spatial resolution of both eyes (the foveae) onto the object of interest, and thus facilitate binocular fusion, resulting in a richer perceptual experience of the selected object. Vergence eye movements are another commonly investigated class of eye movements. Thus we designed an experiment to evaluate the usability of the Tobii EyeX in oculomotor research involving eye vergence.

*Experimental Setup* Observers were positioned in a chin and forehead rest to stabilize head movements, at a distance of ≈1000mm from the screen, i.e. at a vergence distance of ≈3°. Whereas the eye movement measurements described above could be performed using a conventional 2D monitor, the test of vergence eye movements required three-dimensional stimulus presentation. Accordingly, the experiment was conducted with a passive stereo LCD (LG 42LW450A) running at 100 Hz. Observers were required to wear stereoscopic polarized glasses, and a 13P calibration procedure was run monocularly on each subject.

The size of the employed screen (42″) was larger than the screen size (24″) suggested by the manufacturer of the EyeX. However, eye tracking was still possible simply by placing the eye tracker on a stand at 600mm from the observers. To obtain reliable gaze data the device had to be positioned parallel to the screen, as if it were mounted at the bottom of the display.

The experiment was run from a standard PC with an Intel Core i5-2410M CPU @2.30GHz, and 8GB of RAM, equipped with Windows 8.1 OS.

*Stimulus Presentation* The visual stimulus employed to drive binocular fusion was a flat virtual plane positioned in the center of the screen. The stimulus subtended 10° of field of view to ensure full coverage of the area of the field of view that elicits vergence movements (Allison et al. 2004). The plane was textured with 1/f pink noise, which has the same frequency content of natural images (Kretzmer 1952; Bex and Makous 2002; Jansen et al. 2009). A white fixation cross was presented in the center of the stimulus.

The stimulus protocol was conceived to test both divergence and convergence eye movements. The plane was initially presented with 1° of positive disparity, thus requiring observers to fixate at a vergence distance of 4°. Once a subject was properly fixating (which took ≈2s), the stimulus disparity was set to zero, i.e. the plane would be rendered at the actual depth of the screen, thus inducing a divergence movement. This procedure was repeated 50 times, and alternated with a −1° disparity step, which required a convergence movement.

**Fig. 7:**
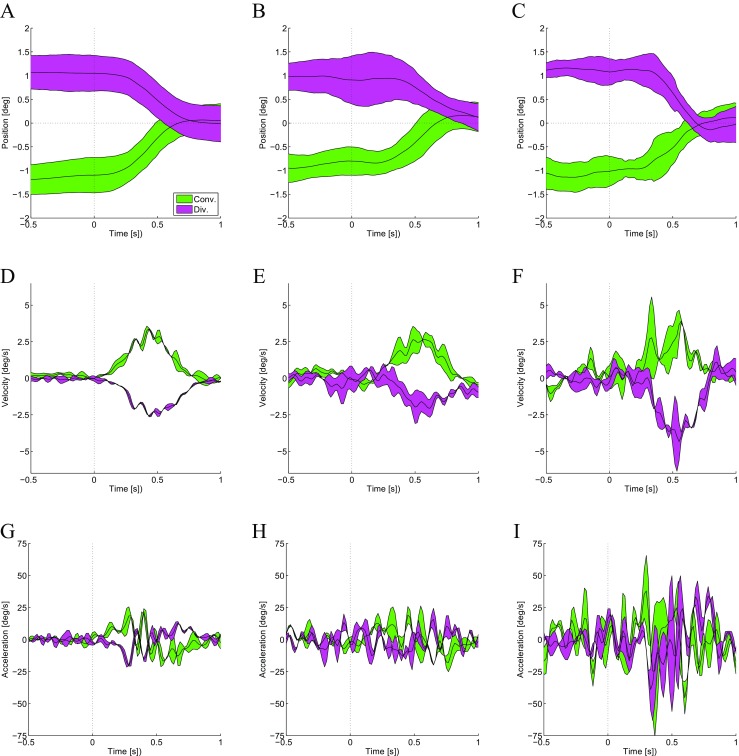
Stereotypical vergence dynamics. Vergence dynamics measured for convergence *(green)* and divergence *(pink)* eye movements in three subjects (columns) for ±1 degree of vergence demand. **a–c** Vergence position as a function of time from vergence target onset. Zero vergence represents the actual depth of the screen. **d–f** Vergence velocity. **h–i** Vergence acceleration. First subject **a,d,g** was author AG and an experienced observer. Second **b,e,h** and third **c,f,i** subjects were inexperienced naive observers. Data are the average from 50 trials. Shaded region represent ±1 SD

*Results* Figure 7 shows the results of our measurements of vergence eye movements in three observers with normal stereo vision. The first subject was an experienced observer (author AG), while the second and third subjects were inexperienced naive observers. Qualitatively we can observe from Fig. 7a-c how the device provides a reliable characterization of the vergence trajectories. The eye movement response delay from stimulus onset was between 100−200 ms, whereas the time required to complete the movement was around 400−500 ms, which is all in good agreement with the literature (e.g. (Hung et al. 1994; Collewijn et al. 1995; Alvarez et al. 2002)). As per the data collected on saccadic eye movements, we measured velocity (Fig. 7 D-F) and acceleration (Fig. 7 G-I) by taking the first and second derivative of the data in Figures 7 A-C using a two point differentiator. Peak velocity was recorded at 3−5 deg/s, while time to peak velocity was between 400−550 ms. The measurements regarding acceleration were noisy, but qualitatively the expected patterns were observed.

#### Fixation distributions in natural scenes

The distributions of fixations in natural viewing provide an interesting tool to study both (top-down) goal-directed (Schötz et al. 2011) and stimulus driven (bottom-up) mechanisms of attention allocation (Henderson 2003). Scan paths, the screen locations our eyes foveate while visually exploring a scene, are indeed often investigated both in neuroscience as well as marketing research.

The following simple experiment has the goal to verify whether the Tobii EyeX is able to provide metrics of eye movement patterns, as well as the distribution of fixations in an image exploration task.

*Experimental Setup* Observers were positioned with their head stabilized by a chin and forehead rest at a distance of ≈700mm from the screen. A stimulus image was displayed for 30 seconds, during which time subjects were instructed to freely explore the scene.

The experiment was performed on a standard PC running Windows 7 Professional, with an Intel Core i7-4700MQ CPU @2.40GHz, and 12GB of RAM, with a 17 inch LCD with 1920 × 1080 resolution running at 60 Hz.

**Fig. 8:**
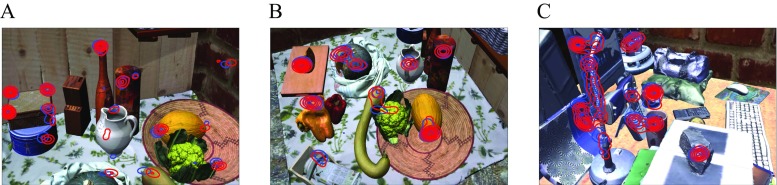
Stereotypical Fixation Distributions in Naturalistic Scenes. Fixational eye movements measured during the free visual exploration (30s) of an image representing a peripersonal workspace. Gaze heatmaps were computed as bidimensional histograms and are shown as contour lines for the *left (blue)* and *right (red)* eye. First subject **a** was author AG and an experienced observer. Second and third **b,c** subjects were inexperienced naive observers

*Stimulus Presentation* The stimuli used for the experiment were 2D rendered images of a 3D virtual workspace representing a kitchen and an office table (Chessa et al. 2009). The workspace was designed to investigate visual behavior in the peripersonal space, and consists of a table (1m × 1m) with ∼ 20 objects positioned at random positions on top of the table (see Fig. 8). The 3D models of the rendered objects were created with a high precision Vivid 910 3D Range Laser Scanner produced by Konica Minolta. The range scanner provides highly accurate 3D meshes (spatial resolution < 1mm) and realistic, high resolution textures (Canessa et al. 2011; Sabatini et al. 2011), that yield a naturalistic perception of the virtual objects.

*Results* Figure 8 shows the results of our measurements of fixation distribution in three observers. The first subject was an experienced observer (author AG), while second and third subjects were naive observers. Fixation maps of visual scene exploration have been computed as bidimensional histograms. These histograms are represented as contour lines for the left (blue) and right (red) eye, separately. The figure demonstrates how the device provides a sensible characterization of the distribution of fixations during the visual exploration task. Furthermore the Tobii EyeX is able to provide other metrics of eye movement patterns, such as the mean fixation duration (1148 ± 780 ms, mean ± standard deviation), and the amplitude (8.9 ± 5.9 deg, left eye, and 8.26 ± 5.85 deg, right eye) and velocity (336.04 ± 234.82 deg/sec, left eye, and 315.96 ± 200.71 deg/sec, right eye) of saccades executed between fixations. The EyeX might thus be employable to study how multiple aspects of visual perception and action interact to determine gaze behavior (Schötz et al. 2011).

The Matlab code used for the proposed experiments is provided in the Appendix A.

## Discussion and conclusions

In this paper we have presented qualitative and quantitative analyses of the characteristics and technical specifications of the Tobii EyeX Controller for its possible use in research applications. We have quantified accuracy, precision, latency and sampling frequency of the device.

### Comparison with other commercial devices

Table 3 presents a comparison between the performances of the EyeX and other eye tracking devices at various price points. The technical specifications reported for the EyeX are those measured in this study.

**Table 3:** Commercial Eye Tracker Comparison

Eye Tracker	Accuracy [*deg*]	Precision [*deg*]	Sampling Rate [Hz ]	Latency [*ms*]	Price Point [*$*]
EyeX	0.5-1	0.25	55	<50	∼ 100
EyeTribe	0.5-1	0.1	30-60	<20	∼ 100
GP3	0.5-1	0.1	60	<50	<1000
myGaze	0.5	0.1	30	<50	<5000
SMI-REDm	0.5	0.1	60-120	<20	<25000
ViewPoint	0.25-1	0.15	90-220-400	<10	<25000
EyeLink 1000	0.25-0.5	0.01	250-500-1000-2000	<10	>25000
Tobii TX300	0.3-0.8	0.1	60-120-250-300	<10	>25000

The technical specifications reported for the other eye trackers are taken from the specification sheets provided by the manufacturers. The accuracy of the EyeX is comparable to that of both low and high-end devices. Conversely, the observed precision of the EyeX device is worse than any of the values reported by the manufacturers of the other devices. The system latency of a gaze contingent display implemented with the Tobii EyeX (< 50ms) is comparable to the the system latency measured with research grade eye trackers (Saunders and Woods 2014) and is acceptable for at least some gaze contingent applications, such as gaze-contingent multiresolutional displays (Loschky and Wolverton 2007). The main difference between the Tobii EyeX Controller and research grade eye tracking technology is the sampling frequency. The Tobii EyeX claims a nominal sampling rate of 60 Hz (which was measured at ≈55 Hz on our setup). Research grade eye trackers instead provide sampling frequencies up to 2000 Hz (e.g. the EyeLink 1000 with 2000 Hz camera upgrade).

### Matlab toolkit

Alongside the characterization of the Tobii EyeX for research purposes, we provide a Matlab Toolkit that allows users to set up eye tracking experiments and employ the EyeX device in an intuitive fashion. The UDP Server Interface which enables communication between the EyeX and Matlab was optimized for quick data transmission and is shown to not affect the system latency. In order to make the Toolkit broadly and easily usable and customizable, we have exploited the functionalities provided by the Psychophysics Toolbox (Brainard 1997; Pelli 1997), which is a widely employed software package for psychophysical research (the original papers describing Psychtoolbox have over 6000 citations on Google Scholar). Because PsychToolbox is such a successful instrument for Psychology and Neuroscience research, our toolbox may enable for PsychToolbox developers to integrate eye tracking directly into their research and we encourage other groups to share code that integrates other low-cost devices directly into the PsychToolbox framework.

The Toolkit we have implemented and made available provides, amongst other features: a simple method to effectively position subjects for optimal eye tracking performance; a reliable and malleable calibration procedure; a rapid and effective method for the online evaluation of the calibration outcome; an intuitive set of functions to collect gaze data. The Matlab Toolkit also includes the procedures to test the device, as well as the methodologies used for the statistical analysis, for a possible benchmark evaluation of other eye tracking devices, in terms of the accuracy, precision and sampling frequency. The experimental code created to run the experiments presented in this paper is included in the Toolkit and is contained within a folder for contributed experimental code. We encourage researchers who employ the Toolkit to submit the code developed for novel experiments implemented with the EyeX. We will periodically update the Toolkit with code shared by the Scientific Community.

### Calibration procedure

We have highlighted the importance of choosing the appropriate calibration procedure, and we have shown that via our proposed calibration routines users can run both monocular and binocular experiments with the appropriate calibration procedures. The calibration procedures we implemented reliably outperformed the TNC. This is likely due, at least in part, to the fact that the TNC was designed to allow users to move their head within a certain range, whereas we performed all our experiments with the subject’s head stabilized in a chin-rest. Allowing head movements is reasonable for consumer applications, but is not optimal for research applications. In carefully designed eye tracking and psychophysical experiments, stimuli must often be systematically presented at precise retinal locations. Thus, the geometric relationships between the observer’s head position and the monitor need to be known and fixed. For this reason, all our calibration procedures require the subject’s head to be stabilized with a chin rest.

### Eye movement data quality

We have performed simple eye movement experiments and have found that the Tobii EyeX can be successfully employed to measure saccadic, smooth pursuit, and vergence eye movements. Furthermore we have found that the EyeX may be employed monitor eye movement behavior in naturalistic visual exploration tasks.

### What might work and what might not

Our evaluation demonstrates that the EyeX is a potentially useful device for multiple research applications. Specifically, we envision this device to be well suited for applications such as fixation compliance and monitoring of simple eye movement parameters. We have so far successfully employed the device for fixation compliance in an array of experiments in which we are measuring: contrast sensitivity, letter acuity and crowding (Maiello et al. 2015; Carroll et al. 2016); motion discrimination (Maiello et al. 2015; Chessa et al. 2016); reading speed (Bex et al. 2015); illusory object completion (Ayeni et al. 2015); retinal disparity patterns experienced by an observer (Gibaldi et al. 2015); active binocular fixation strategy in 3D environments (Gibaldi et al. 2016). These data have been successfully collected from expert psychophysical observers, undergraduate students, and even clinical populations. We further plan on employing the EyeX tracker to measure vergence eye movements when assessing interactions between binocular fusion and spatial frequency (Kwon et al. 2015; Gibaldi et al. 2016), and to validate modeled vergence behavior (Gibaldi et al. 2010; Gibaldi et al. 2012). An intriguing possibility would also be that of employing low cost eye trackers such as the EyeX in continuous target-tracking tasks to rapidly measure visual function (Bonnen et al. 2015). The small dimensions and portability of the device also make it a good candidate for field experiments where large and expensive devices (such as the EyeLink which requires a dedicated PC) are not easily employed.

However, while the low-cost nature of this device makes it an optimal candidate for gathering preliminary data and pilot testing novel ideas, the low sample frequency and limited precision of the device are not yet sufficient for all research applications. The temporal resolution of the EyeX is clearly insufficient to study perisaccadic visual perception (Ross et al. 2001). The precision of the device is also unlikely to be sufficient in measuring fine oculomotor adjustments such as those observed in saccade adaptation paradigms (Pelisson et al. 2010). Clearly the measurement of tiny microsaccadic eye movements (for a recent review see (Rolfs 2009)) is well beyond the capabilities of the device.

### Final remarks

We have thus reviewed the strengths and limitations of the Tobii EyeX eye tracker. Overall, we are encouraged that the Tobii EyeX, together with other emerging low-cost devices (Dalmaijer 2014; Ooms et al. 2015) and recent developments in web-cam based eye tracking (Xu et al. 2015), represents a meaningful step towards a widespread adoption of eye tracking technology, both in commercial and research applications.

## A Matlab toolkit

The implemented software Toolkit for Matlab communication is released by the authors **for research purposes only**, and can be downloaded at:

https://sourceforge.net/p/matlabtoolboxeyex.

For an explanation of the source code relative to the procedures used to evaluate accuracy, precision and sample frequency of Tobii EyeX, and how to adapt them to other eye tracking devices, refer to the Wiki page of the Matlab Toolkit.

The Toolkit also includes the sample code to replicate the proposed experiments for saccade, smooth pursuit, vergence and visual exploration. The software structure has been conceived in order to easily extend the Toolkit with other experiments shared by the Scientific Community. For further information and for the software guidelines, refer to the Wiki page of the Matlab Toolkit.

### UDP server interface

The core of the Matlab Toolkit consists of a UDP Server Interface which allows communication between the EyeX controller and Matlab. Specifically, the server application sends commands from Matlab to the Tobii EyeX Engine and sends gaze data from the Tobii EyeX Engine to Matlab.

### Basic connection functions

The tobii_connect function launches the server application, connects the Tobii EyeX on the IP address, and opens a UDP port for data transmission and receiving.

The tobii_command function transmits and receives data and commands to/from the server. The function can transmit commands to: 1) initialize the device (command ='init'), 2) begin gaze data acquisition and recording of raw data to a txt file (command ='init', arg ='file_name'), 3) acquire a single datum from the device (command ='read'), 4) interrupt gaze data acquisition (command ='stop').

The tobii_close function closes the server application and the UDP port.

The following tobii_get⋆ functions are dedicated to receive real-time data from the device and may be employed instead of the general purpose tobii_command function. The Matlab Toolkit thus provides direct access to: the left and right eye gaze position of the screen, in millimeters (tobii_getGPM) or normalized to the screen size (tobii_getGPN), the actual 3D position of the two eyes with respect to the screen center, in millimeters (tobii_getEPM) or normalized to the trackbox size (ttobii_getEPN), a combination of the normalized eye and gaze position (tobii_getGPN_EPN), or the current timestamp (tobii_getTime).

Since these functions are similar to each other in terms of input and output parameters, we only report selected examples.

### Routines

Since the positioning of a user in front of the monitor affects the accuracy of the device, the Position Guide function, similarly to the one implemented in the EyeX Engine, provides feedback to the users on their current position with respect to the optimal positioning for eye tracking. The routine provides a graphical interface, implemented through the Matlab Psychotoolbox, which visualizes the user’s position with respect to the center of the screen. The user’s eyes are rendered in red when the user is too far or too near to the device. The rendered eye size changes as the user moves closer or farther from the display. When the user is correctly positioned near the center of the trackbox, the eye color turns green, allowing for a rapid and effective positioning of the user in front of the screen.

The CalibrationProcedure function is the core of the calibration procedures, as described in Section 3. The function takes as input an Nx2 vector containing the N normalized coordinates (X and Y) of the targets to be used for calibration, and returns two structures, for the left and the right eye, containing the calibration fit functions for the X and Y gaze estimation. In the present work we investigated whole screen device calibration, but the procedure can be easily tuned to the requirements of different experiments or applications. For instance, in the vergence experiment presented in Section 3, solely a small central portion of the monitor contained the visual stimuli. Accordingly, the calibration procedure was modified and consisted of eight targets positioned on a circle of radius 2.5°, plus one in the exact center of the screen.

The CalibFit function is used by CalibrationProcedure to fit the gaze estimation error. The error, computed for each target on the X and Y screen position, is input to the function along with the targets’ position. The function fits a biharmonic function to the data and outputs the surface that best describes the error over the screen area. Other types of fitting (lowess and cubic interpolation) are available.

The tobii_getGPNcalib function is a modified version of tobii_getGPN. In addition to acquiring the current gaze point normalized to the screen size, it applies to the acquired gaze point the calibration surfaces computed by CalibFit and returns, in real time, the calibrated left and right gaze point.

The tobii_GPNcalib function is a modified version of tobii_getGPNcalib, dedicated to calibrating acquired data offline. The function takes as input a 2*x* *N* array of normalized gaze points and applies to these points the calibration functions computed by CalibFit.

The CalibrationCheck function is a modified version of PositionGuide, conceived to provide a visual check of the calibration achieved. The routine provides a graphical interface, implemented with Matlab Psychotoolbox, that visualizes the measurement error of the device. All the targets used for calibration are shown on the screen. When the user is fixating one of the targets, the target turns red and the left and right rendered eyes change color, depending on the measurement error: green for small errors and red for large errors. A number of factors may affect the calibration, such as: level of compliance to task instructions, variable environment illumination, glasses or contact lenses, makeup, eye physiology, etc. Consequently, CalibrationCheck is a useful and easy-to-use tool to verify the result of the calibration procedure, and possibly run it again, in order to avoid collecting biased or wrong data. The evaluation is provided separately for the left and right eye.

### Sample code

Sample code is provided within the Toolkit. Separate subfolders within the Toolkit contain the code for each of the experiments presented in this paper. The templates can be easily modified by any user for their specific purposes. The calibration target positioning and the experimental stimulus presentation can be easily modified within the script. As an example of how to employ the EyeX, in this Appendix we report and describe solely the code for the visual exploration task. For other sample code please refer to the wiki page of the Matlab Toolkit.

The script EXAMPLE_FIXATION provides MATLAB code used for the measurement of visual exploration eye movements on an image presented in Section 3. After having activated and initialized the Tobii EyeX, a uniform gray window is opened via Psychotoolbox. The window is first employed to perform a 9PC, and next to display the image to be explored. The left and right eye position measured during the experiment are used to compute a bidimensional histogram which graphically represents the distribution of fixations within the image (see Fig. 8).
